# Hydrolysed Formulas in the Management of Cow’s Milk Allergy: New Insights, Pitfalls and Tips

**DOI:** 10.3390/nu13082762

**Published:** 2021-08-12

**Authors:** Enza D’Auria, Silvia Salvatore, Miriam Acunzo, Diego Peroni, Erica Pendezza, Elisabetta Di Profio, Giulia Fiore, Gian Vincenzo Zuccotti, Elvira Verduci

**Affiliations:** 1 Department of Pediatrics, Vittore Buzzi Children’s Hospital, University of Milan, 20154 Milan, Italy; miriam.acunzo@unimi.it (M.A.); erica.pendezza@unimi.it (E.P.); elisabetta.diprofio@unimi.it (E.D.P.); giulia.fiore2@studenti.unimi.it (G.F.); gianvincenzo.zuccotti@unimi.it (G.V.Z.); elvira.verduci@unimi.it (E.V.); 2 Department of Pediatrics, Ospedale “F. Del Ponte”, University of Insubria, 21100 Varese, Italy; silvia.salvatore@uninsubria.it; 3 Department of Clinical and Experimental Medicine, Section of Pediatrics, University of Pisa, 56126 Pisa, Italy; diego.peroni@unipi.it

**Keywords:** cow’s milk allergy, cow’s milk protein-based hydrolysed formulas, vegetable protein based hydrolysed formulas, immune modulation, amino-acid based formulas

## Abstract

An allergy to cow’s milk requires the avoidance of cow’s milk proteins and, in some infants, the use of a hypoallergenic formula. This review aims to summarize the current evidence concerning different types of hydrolysed formulas (HF), and recommendations for the treatment of IgE- and non-IgE-mediated cow’s milk allergy and functional gastrointestinal disorders in infancy, for which some dietary intervention and HF may be of benefit to both immune and motor mechanisms. Current guidelines recommend cow’s milk protein (i.e., whey or casein) extensively hydrolysed formula (eHF) as the first choice for cow’s milk allergy treatment, and amino acid formulas for more severe cases or those with reactions to eHF. Rice hydrolysed formulas (rHF) have also become available in recent years. Both eHF and rHF are well tolerated by the majority of children allergic to cow’s milk, with no concerns regarding body growth or adverse effects. Some hydrolysates may have a pro-active effect in modulating the immune system due to the presence of small peptides and additional components, like biotics. Despite encouraging results on tolerance acquisition, evidence is still not conclusive, thus hampering our ability to draw firm conclusions. In clinical practice, the choice of hypoallergenic formula should be based on the infant’s age, the severity, frequency and persistence of symptoms, immune phenotype, growth pattern, formula cost, and in vivo proof of tolerance and efficacy.

## 1. Introduction

Cow’s milk (CM) protein is one of the most common food allergies (FAs) in infancy, with a region-dependent prevalence of 2–3% [1,2,3,4].

The immune reaction to CM proteins can be IgE-mediated, non-IgE-mediated, or mixed. It can also differ in its timing of symptom onset and organ involvement. Immediate reactions occur from within a few minutes to no more than two hours after exposure to the offending food, while delayed reactions can occur up to 48 h or even a week later. The former are classified as IgE-mediated, while the latter are generally non-IgE-mediated [1].

Clinical manifestations of cow’s milk allergy (CMA) vary greatly in type and severity, making it one of the most difficult food allergies to diagnose [4,5].

Most symptoms of non-IgE-mediated FAs involve the digestive tract. Indeed, non-IgE-mediated FAs include food protein-induced allergic proctocolitis (FPIAP), food protein-induced enterocolitis syndrome (FPIES) eosinophilic disorders, including eosinophilic esophagitis (EoE), and a heterogeneous group of non-IgE-mediated disorders more commonly recognized as gastrointestinal motility disorders [6,7].

The diagnosis of both non-IgE and mixed FAs is mainly clinical and is often challenging.

In contrast with IgE-mediated FAs, the onset of symptoms is often delayed and they may follow a chronic course, making the association with the allergen less evident [8]. Furthermore, no laboratory tests can assist in the diagnosis. Thus, in most cases, diagnosis relies on symptom resolution once the suspected food is eliminated and symptom reappearance when it is reintroduced [9].

Of note, some children who tested negative for a specific IgE may become IgE positive over time [1].

The oral provocation test remains the gold standard for the diagnosis of both IgE- and non-IgE-mediated allergies, with the exception of cases characterized by a high pre-probability test, e.g., in cases of anaphylaxis or clinical history suggestive of immediate reaction and positive skin prick test or serum specific IgE [10].

Since the strict avoidance of CM proteins is currently the safest strategy for managing CMA, in the absence or insufficiency of breast milk, the child must be fed with a formula adapted for CMA dietary treatment, e.g., a hypoallergenic formula.

The aim of this review is to summarize the current evidence regarding different types of hydrolysed formulas, with a focus on immune modulation and nutritional values, and guideline recommendations in the treatment of IgE- and non-IgE-mediated CMA.

A comprehensive search was conducted using the electronic databases MEDLINE via PubMed (www.pubmed.gov, accessed on 8 May 2021) and Embase databases (www.embase.com, accessed on 8 May 2021).

The following keywords were used: hypoallergenic formula, hydrolysed, hydrolysed formula, extensively hydrolysed formula, amino acidic formula, rice-based formula, soy-based formula, cow’s milk allergy (CMA), tolerance or nutritional value or growth in CMA, allergic proctocolitis, food protein-induced enterocolitis syndrome, eosinophilic esophagitis, gastrointestinal, motility disorders, and immune modulation.

## 2. Hydrolysed Formulas: New Regulations and Composition

The term “hypoallergenic formula” does not have a univocal meaning across the world.

In the USA, as defined by the American Academy of Pediatrics, the term indicates a formula that is tolerated by at least 90% of children with proven CMA, with a 95% confidence interval, when given in prospective randomized, double blind, placebo-controlled trials [11]. In Europe, on the other hand, it refers generically to a formula containing hydrolysed protein, thus having a reduced allergenicity [12].

The Commission Delegated Regulation (EU) 2016/127 sets out micro and macronutrients contents for hydrolysed formulas (HF), whether from animal (whey and casein proteins) or vegetable sources (soy proteins), in the first year of life for oral administration [13].

Table 1 shows the nutrient composition of both hydrolysed and soy formulas required for infancy and the follow-on period. The main differences between the two types of formula are the protein range for 100 kcal, which is lower in the animal source formula, and the amount of iron and zinc, which is higher in the soy formula. HF are manufactured through various processes, including enzymatic hydrolysis, heat-treatment, and ultrafiltration. These processes are needed to decrease, and ideally remove, the allergenicity of the resulting formula [14].

HF that are currently on the market are different in terms of protein source (i.e., animal or vegetable), methods and degree of hydrolysis, and additional components (i.e., pre- and probiotics, and thickener components) that may influence the clinical results [15,16].

These variable characteristics mean that different brands cannot be compared. Based on the degree of enzymatic hydrolysis, HF are classified as partially hydrolysed formulas (pHF) or extensively hydrolysed formulas (eHF).

In general, pHF contain peptides with a molecular weight <5 kDa, ranging from 3 to 10 kDa, while eHF are composed of more than 90% short peptides <3 kDa (mostly <1.5 kDa) and free amino acids [17,18].

According to European guidelines, “hypoallergenic” formulas intended for the dietary treatment of CMA include both eHF and amino acid formulas (AAF), which are the only “anallergenic” options, as they contain a mixture of free amino acids [19].

In addition to hydrolysed cow’s milk-based formulas, hydrolysed vegetable protein-based formulas, characterised by a different degree of hydrolysis (i.e., rice hydrolysed based formulas and soy hydrolysed based formulas), are also available on the CMA market.

With regard to rice formulas, the European Food Safety Authority stated that the total arsenic content in rice-based infant formula is 0.158 mg/kg dry product, assuming that 70% of the total arsenic is inorganic [20]. As such, in 2016, the Commission Delegated Regulation (EU) 2016/128 stated that the maximum rice inorganic arsenic content for food ingested by children under the age of 3 is 0.10 mg/kg (two times lower than that applied to white rice products) [13]. In regard to the arsenic content of rice formulas, safe limits were also set out by the European Society for Paediatric Gastroenterology Hepatology and Nutrition (ESPGHAN) nutrition committee recommendations in 2015 [21]. However, in the majority of commercialised rice hydrolysates, arsenic content is not specified on the label.

Soy infant formulas are also available for CMA treatment. However, they are not recommended for infants younger than 6 months of age nor for infants with gastrointestinal CMA-related symptoms, due to more adverse reactions having been observed in those groups [22,23,24].

## 3. Hydrolysed Cow’s Milk and Vegetable Protein-Based Formulas: Tolerance, Nutritional Value, and Palatability

Both eHF and pHF contain a wide range of peptides, but differ according to their molecular weight profile.

The primary aim of CMA treatment is symptom resolution via the use of a hypoallergenic formula.

Hypoallergenicity is achieved through the destruction of protein epitopes, which are responsible for IgE binding [25].

As stated by the American Academy of Pediatrics, eHF should only contain peptides that have a molecular weight of <3 kDa [17]. However, there is no conclusive evidence that this threshold is sufficient to prevent allergic reactions in infants with CMA [1].

Although less than pHF, residual allergenicity is also present in eHF, particularly those containing peptides of >3.5 kDa [26].

In fact, allergic reactions were described with eHF in selected cases [27].

For this reason, the British Society for Allergy and Clinical Immunology (BSACI) guidelines suggest that eHF containing the greatest percentage of peptides < 1000 Da are preferable [4] for CMA treatment.

Due to the presence of large peptides that may trigger reactions in children allergic to cow’s milk, pHF, based on CM proteins, are not recommended for the treatment of CMA [1,19].

Only AAF exclusively provide nitrogen equivalent proteins as free amino acids, which cannot lead to any immune stimulation. Therefore, they are the only formulas that are totally anallergenic [19].

Rice-based HF are marketed, with long-term use, in Italy, Spain, and France [28]. Rice-based HF are tolerated by at least 90% of CMA-allergic children, as recently addressed [29]. Notewothy, most of the clinical studies assessing rice-based formula were conducted on children with IgE-mediated CMA [30,31,32]. Only two studies were conducted on both IgE-mediated and non-IgE-mediated CMA, both of which confirmed the high tolerability of this formulation even in non IgE CMA [26,33].

Regarding nutritional aspects of eHF, rice-based formulas, and AAF, the main concern involves the rate of peptides and amino acid content. The protein source of an HF should have almost 50% essential amino acids, represented by branched chain amino acids and valine [34].

Amino acids can have different rates of digestion, metabolism, and absorption. The rate of entry of amino acids from peptides will be faster than that of intact dietary protein and may even be faster than that of free amino acids. This high rate of transport can have consequences on the metabolic processes and utilization of protein hydrolysates; nevertheless, no significant adverse effects from the ingestion of protein hydrolysates have been reported [35]. HFs contain 100 times more free amino acids, mainly branched chain amino acids and glutamate [36], than standard formulas [37].

Regarding the use of AAF, it is essential to achieve a balance between the amino acids ingested (to avoid an excessive increase in N excretion) and the energy intake (through glucose), to promote protein anabolism. A ratio of 3–4.5 g of protein (equivalent)/100 kcal was proposed, corresponding to 12–18% of the total energy to achieve this result [38].

The amino acid profile of rice proteins naturally differs from bovine milk proteins: although it is rich in essential amino acids, threonine, lysine and tryptophan are contained in lower quantity compared to breast milk.

Hence, rice HF are generally supplemented with these three amino acids [39]. Rice protein hydrolysis is required to improve water solubility and digestibility [30]. Soy formulas may have a lower absorption of minerals and trace elements due to their phytate content. The concern about the presence of isoflavonoid has also limited their use and, for this reason, newer formulations have greatly reduced its content. Currently, soy-based formulas are all supplemented with amino acids (methionine, taurine, and carnitine) [40].

A substantial number of clinical studies have evaluated the safety and efficacy of different hydrolysed formulas in the treatment of CMA. They are summarized in Table 2.

In addition, reviews and meta-analysis have also been conducted.

In 2020, Strózyk et al. performed a systematic review of the use of HF in the treatment of CMA by comparing 15 European clinical trials [41].

The authors concluded that all evaluated eHF were well tolerated by the majority of children with CMA, with no concerns regarding growth or other adverse effects.

Normal growth was also reported in another meta-analysis comparing AAF plus symbiotic vs. AAF alone [42].

However, it should be noticed that most of the studies considered small samples of children and treatment for relatively short periods of time, with only one study in which a two-year follow-up was conducted.

With regard to rice hydrolysed formulas, studies have been performed mostly on patients with an IgE-mediated allergy [31,32].

To date, no data exist on the use of rice hydrolysates in cases of non-tolerance to eHF, as an alternative to AAF. Finally, there is a lack of randomised clinical trials comparing the efficacy of rice hydrolysate with soy-based formulas or other hydrolysates.

So far, the only existing long-term follow-up study regarding HF is the German Infant Nutritional Intervention (GINI) study. In this study, 1840 high-risk infants fed with pHF, eHF (casein or whey), standard formula, or breast milk were followed up until 10 years of age, with no significant differences identified between the different groups in terms of body mass index, weight, or height z-scores [43]. This study is of considerable importance due to both the high number of patients and the long-term follow up, since, from a clinical safety perspective, this is the most reliable method to assess the long-term effect of formulas.

Another important aspect to be considered is the palatability of HF, which may influence compliance with the avoidance diet. Overall, the taste of HF is often bitter and not very palatable, due to their particular composition [44,45,46]. Generally, The introduction of HF is easily accepted in children <4–6 months of age as taste is influenced by learning and habits [47]. A practical strategy to favour formula acceptance is by offering the same formula at least 8–10 times, since repeated exposure is the most effective way of promoting acceptance of new foods in infants and toddlers [48].

## 4. Immune Modulation by Hydrolysed Formulas

Beyond the role of symptom relief, hydrolysates may also have a pro-active effect in modulating the immune system through different mechanisms [16].

Hydrolysates were found to strengthen the epithelial barrier through the small peptides derived from HF, which in turn increased the expression of gene coding for tight junction proteins. Improved barrier function decreases absorption of the antigen and its contact with intestinal immune cells, thus reducing allergic symptoms [49].

They also modulate T cell differentiation and decrease inflammation by skewing the differentiation of T cells from a Th2 subtype towards Th1 or Treg, and by stimulating tolerogenic responses in antigen presenting cells [50].

Finally, by enhancing regulatory B cells, they promote the secretion of regulatory cytokines (such as IL-10) and decrease pro-inflammatory markers (such as COX-2, NF-kB, and IL-8) [50,51,52].

These effects have been demonstrated through in vitro and ex vivo studies [53,54,55,56]. Peptides that exert an immunomodulatory action are generally very small, ranging from 2 to 20 amino acids; however, some heavier peptides (with a molecular weight of >1000 Da) from soy and whey protein hydrolysates have also shown the same property [57].

In vivo studies investigating the role of different formulas on tolerance acquisition have been conducted, with no conclusive results [33,43,58,59]. Of note, only a few immunomodulatory peptides have been identified thus far. Further research should target the recognition of all immunomodulatory peptides and their immune effects in humans.

Some HF were added with specific strains of probiotics, which may themselves act on immunomodulation, in addition to the small peptides.

It is known that Lactobacillus rhamnosus GG (LGG) play a role in the regulation of some genes involved in the immune and inflammatory response by altering the presence of some cytokines involved in IgE- and non-IgE-mediated milk protein allergy [60,61].

A number of studies have evaluated the effect of HF supplemented with various probiotics, such as LGG or Lactobacillus casei CRL431/B lactis Bb12, in order to assess the clinical course of allergic manifestations and tolerance acquisition in allergic children [62,63,64,65,66,67,68].

Despite encouraging results, most of the evidence was derived from the same group of researchers and, thus, requires further confirmation thorough randomised controlled trials, before any firm conclusions can be drawn.

Of note, AAF, although considered the safest dietary strategy for infants with severe CMA, may not be able to promote tolerogenic effects, mainly due to the absence of small peptides [1,33]. Paparo et al. showed the absence of effects on the intestinal barrier, Th1/Th2 cytokine response, and activation of Tregs in vitro [56].

With regard to symbiotics, a randomized trial, which recruited 110 children diagnosed with CMA, administered either an AAF or AAF with symbiotics (oligofructose, long-chain inulin, acidic oligosaccharides, and Bifidobacterium breve M-16V) to trial subjects. The authors found no differences in growth pattern, allergic symptoms, or even stool characteristics.

Overall, despite some promising results, it is currently not possible to recommend the use of formulas with added probiotics, prebiotics, or symbiotics to favour tolerance acquisition, as the evidence is still scarce and conflicting [16,69].

Likewise, the most effective strains, dosages, or optimal duration of treatment are still yet to be determined [16,69].

## 5. Hydrolysed Formulas in CMA Treatment: Recommendations

It is mandatory to identify an alternative milk for non-breastfed infants and children <2 years old, while, in children from 2 years of age, adequate energy, protein, calcium, and vitamins can be obtained from other sources [4].

The European guidelines and ESPGHAN Guidelines recommend cow’s milk protein-based eHF as first line treatment for children with CMA (Table 3).

Rice HF represents a valid alternative in clinical practice for treating infants with CMA and, as such, they are formally included in some guidelines, although with different indications [1,28].

In the Diagnosis and Rationale for Action against Cow’s Milk Allergy (DRACMA) guidelines, rice HF are considered equivalent to cow’s milk protein-based eHF in the treatment of infants with CMA, in countries where they are available. In contrast, ESPGHAN guidelines only recommend rice HF for children in vegan families, or those who refuse or cannot tolerate casein or whey eHF. Soy-based formulas have mostly been used in children with IgE-mediated forms, while data on large populations of children with non-IgE-mediated allergies are lacking.

Guidelines recommend the use of soy protein-based formulas as an alternative to eHF only for children >6 months and once tolerance for soy protein has been established [1,4].

Although most children allergic to cow’s milk proteins tolerate eHF, a percentage of them need to be introduced to AAF, the only truly anallergenic alternatives. These formulas can be used as a first choice in case of severe symptoms of CMA at the onset, such as anaphylaxis, multiple food allergy with growth faltering, in severe forms of allergy like FPIES, or as a second choice in the case of non-tolerance to eHF (Table 3).

The “perfect” hydrolysate would be hypoallergenic (in vivo), with no cross-reactivity, good palatability, a balanced amino acid content and lipid profile and low cost.

Since such a formula does not exist, it is important to choose the appropriate formula for each child, taking into account a number of different factors, such as age, clinical symptoms, sIgE repertoire i.e., CMA endotype, palatability, availability, and cost-efficacy ratio [19,70].

Main strengths and weaknesses of the different hydrolysed formulas are summarized in Table 4.

### 5.1. Hydrolysed Formulas in IgE mediated CMA

IgE-mediated CMA may present different immunophenotypes, although most sensitized infants present sIgE to multiple CM proteins [71].

IgE-mediated reactions involve, in order of frequency, the skin, the gastrointestinal tract, and the respiratory system [4].

In children with severe IgE-mediated CMA, such as anaphylaxis, growth faltering, or multiple food allergies, the guidelines agree on the use of AAF as the first line of treatment, while for those with less severe symptoms, such as isolated cutaneous symptoms, urticaria, or immediate vomiting, CM eHF is considered the first choice [19,28].

However, rice protein-based formulas may also be considered a first step therapeutic option as an alternative to eHF [28]. Although not formally addressed by the guidelines, rice-based formulas may be considered a valid alternative to CM-based HF in cases of immunophenotypes characterised by multiple sensitisation to both whey and casein.

There are no data as of yet on the use of rice HF as a second line of therapy in children who do not tolerate CM eHF [29].

### 5.2. Hydrolysed Formulas in Non-IgE CMA and Mixed Forms with Gastrointestinal Symptoms

Non-IgE-mediated food allergies presenting with GI symptoms constitute about 50% of CMA, and include different clinical entities with a wide spectrum of severity, ranging from mild or moderate forms, such as food protein induced enterocolitis (FPIAP), to more severe acute clinical manifestations, like food protein induced enterocolitis (FPIES) and eosinophilic gastroenteropathies [8,72].

Briefly, the following sections will discuss issues concerning the dietetic management of these forms, mostly focusing on the use of HF and AAF. A more comprehensive review of pathogenetic and clinical aspects of non-IgE CMA is beyond the scope of the present review as they have been addressed in other publications [6,72,73].

#### 5.2.1. Food Protein Induced Allergic Proctocolitis [FPIAP]

FPIAP is a recognized cause in 60% of children with haematochezia. In non-breastfed infants, the introduction of an eHF usually allows symptoms to be resolved, with only a small percentage of patients requiring the introduction of an AAF due to persistent stool bleeding, if associated with other complications, such as anaemia [8].

Trials showed that in infants with allergic proctocolitis, casein-based eHF supplemented with LGG induced a reduction in faecal blood and faecal calprotectin after one month [74,75]. However, the potential clinical impact of these findings needs to be further evaluated.

#### 5.2.2. Food Protein Induced Enterocolitis [FPIES]

Non-IgE-mediated enterocolitis, or FPIES, is considered a rare condition, which may also be underdiagnosed [8].

In most cases, patients with FPIES react to a single food allergen and CM are considered the main allergens responsible for these forms, although other foods may be involved in the allergic mechanism [73].

Breastfeeding should always be encouraged and supported. However, when breastfeeding is not possible or insufficient, the most appropriate hypoallergenic formula should be chosen, but which formula to use as a first line choice remains under debate. European guidelines [1,4,19] recommend AAF instead of eHF, especially in children with severe symptoms, such as chronic FPIES. On the other hand, the U.S. guidelines [76], the DRACMA Guideline [28], and the International Consensus guidelines for the diagnosis and management of FPIES, published in 2017 [73], recommend that infants with CM or soy-induced FPIES may use a casein-based eHF as a first choice, while only 10–20% may require AAF.

As a practical approach, starting treatment with eHF was suggested with the subsequent replacement of an AAF in patients who do not show satisfactory symptom relief or growth recovery within two weeks [77]. AAF should therefore be considered in order to support infant nutrition, reduce symptom severity, and speed up hospitalization time [78,79].

#### 5.2.3. Eosinophilic Diseases

Non-EoE eosinophilic gastrointestinal diseases, eosinophilic gastroenteritis, eosinophilic gastritis, and eosinophilic colitis are a group of rare diseases, which are classified on the basis of intestinal tract eosinophilic infiltration [80].

The role of diet therapy in the treatment of GI diseases caused by eosinophils is still under debate.

The treatment of a rigorous elemental diet was shown to be effective in 75% of infants affected by eosinophilic colitis and gastroenteritis [81], while Dellon et al. found a lower percentage in a study involving both children and adults [82].

Eosinophilic esophagitis is a disorder characterised by chronic relapsing symptoms of oesophageal obstruction and dysfunction, and severe eosinophilic infiltration (≥15 eosinophils per high power field in at least one oesophageal biopsy) [83,84]. CM is recognized as the main triggering food in EoE in about 75% of children and is even higher in infants [85].

An elemental diet with an elemental formula was demonstrated to be highly effective both in resolving clinical symptoms as well as inducing histologic remission in paediatric patients affected by EoE [86,87]. In one small study of 17 adult patients, 88.2% achieved and maintained remission when supplemented with extensively hydrolysed whey protein formula for eight weeks, with no difference in the endoscopic appearance at baseline or blood eosinophils count and no personal or family history of allergy [88]. However, more research on HF is needed and AAF is currently the recommended formula for patients with EoE. There are currently no data on the possible efficacy of eHF in eosinophilic esophagitis in infants.

## 6. Hydrolysed Formulas in Gastrointestinal Motility Disorders

In addition to the abovementioned forms of CMA, HF was considered in selected patients with functional gastrointestinal disorders or more recently defined gastrointestinal motility disorders [89,90] and particularly in infants with gastroesophageal reflux (GER) and colic.

Infants with CMA or GER and GER-disease (GERD) may present similar gastrointestinal, respiratory, and general symptoms including regurgitation, vomiting, coughing, wheezing, crying, irritability, feeding and sleeping problems, and failure to thrive. Likewise, a response to HF may occur in these conditions because of immunological and motor related mechanisms in CMA or by improving gastric emptying in GER and GERD [16]. Thus, a reaction to a CM oral challenge with only gastrointestinal symptoms can occur beyond immune factors. In subjects with an IgE-mediated food allergy, the smooth muscle contractility is mostly affected by interleukin (IL)-4, IL-13 cytokines, and transforming growth factor-beta [91], while in non-IgE-mediated CMA, motility is impaired by tryptase released by mast cells migrating and interacting with nerves [92]. The lack of a specific symptom and a gold standard for diagnostic tests of GERD and non-IgE-mediated CMA, the overlap with other functional and organic conditions, and the frequent natural resolution of symptoms in the first year of life, make the diagnosis and discrimination between CMA, GER, and GERD an ongoing challenge in infants [92].

Fifty infants with persistent unexplained crying, vomiting or food refusal were recently, extensively investigated via allergy tests, oral food challenge, oesophageal pH-impedance monitoring, 13 C-octanoate breath testing, dual-sugar intestinal permeability, faecal calprotectin, and serum vitamin D level. Fourteen infants (28%) had a final diagnosis of CMA, but no test showed a high predictive value. In infants on a CMA elimination diet (with AAF) there was significant improvement in GERD symptoms, oesophageal clearance and impedance baseline, all of which are considered as indirect parameters of oesophageal integrity [93].

A number of studies examined the relationship between CMA and GER or GERD in infants with a wide range of association (16–56%) depending on population recruitment, diagnostic criteria, outcomes, dietary intervention, and follow-up data [94].

In 1996, Iacono et al. first reported an improvement of symptoms attributed to GERD in 85/204 (42%) infants when fed an HF and with relapse on challenge. Approximately half (45%) of the enrolled population had positive allergy tests, and the group with GER and CMA was significantly associated with the presence of diarrhoea or atopic dermatitis [95].

In 2002, Garzi et al. found a delayed gastric emptying in 10/10 infants with GER symptoms (compared to healthy controls), with a significant improvement of both gastric emptying time and symptoms when fed eHF, particularly in subjects with positive IgE tests [96]. In 2013, Vandenplas introduced a CM score (originally called SBS and then CoMiSS) assessing two extensive HFs with probiotics. All clinical scoring criteria (including crying, regurgitation, stool consistency, eczema, urticaria, and respiratory symptoms) were reduced from baseline to one month evaluation during the dietary intervention [44]. A similar drop in clinical symptoms was obtained by the same group in another population of 40 infants with a positive CM challenge when fed a rice eHF [97].

In 72 infants with persistent gastrointestinal and additional respiratory or dermatological symptoms, the clinical efficacy of a thickened and non-thickened casein eHF was tested [97]. Regurgitation was significantly reduced in all infants after one month of dietary intervention (6.4 ± 3.2 episodes/day at enrolment to 2.8 ± 2.9 after one month, *p* < 0.001). The thickened HF reduced the daily number of regurgitation episodes slightly more than the non-thickened HF (−4.2 ± 3.2 vs. −3 ± 4.5, *p* = 0.24), with a similar difference comparing the group with positive and negative challenges (−4.5 ± 4 vs. −2.8 ± 3.7, *p* = 0.20) [98]. When testing the same formulas in 77 infants with suspected CMA and troublesome regurgitation and or vomiting of more than five episodes/day, the authors found that decreased regurgitation was present in both eHF, but with a higher degree when using the thickened formula after one month of intervention [99].

Interestingly, in another group of 30 infants with CMA proven by a double-blind food challenge (70% with positive IgE tests) and using a casein-based, thickened eHF, both regurgitation and crying clinical scores significantly decreased after only 14 days on the diet [100].

We evaluated 47 infants (median age three months) who were put on a cow’s milk free diet (62% on an extensively HF) due to persistent unexplained gastrointestinal symptoms [16]. In 35/47 (75%), regurgitation was reported at least three times per day at enrolment and these episodes halved when on a CM-free diet. A clinical score also assessed that both frequency and volume of regurgitation decreased from 2.0 to 0.6 when on the diet [16].

In the first few months of life, functional gastrointestinal disorders occur in up to 50% of subjects, with regurgitation and infantile colic representing the two most common conditions that often spontaneously resolve or improve by 6 to 8 months of age.

According to Rome IV criteria, infant colic is characterized by the presence of unexplained “crying” or “irritability” in the absence of any organic cause [101]. Altered intestinal motility, psychological and behavioural disturbances, hyperalgesia, and intestinal dysbiosis were also proposed as underlying pathogenic mechanisms for which there are a variety of treatment options [102,103]. Prolonged unexplained and inconsolable crying in infancy is often attributed to both CMA and GER disease, although these two conditions were demonstrated in only a minority (5–10%) of subjects [94]. CMA should be suspected in cases of particularly severe and persistent symptoms, and in the presence of concomitant atopic dermatitis, gastroesophageal reflux disease, severe irritability, or refusal of food [104].

In 1988, in a small trial infants with unexplained crying for at least 2 h per day were entrolled; 10 infants started a CM-free diet (with 7 put on a casein eHF, and 3 continued breastfeeding with a maternal elimination diet). Nine days later, the crying decreased from 3.19 ± 0.69 h/d to 2.03 ± 0.7 h/d (*p* = 0.01), but less than in the group that was provided parental education and counselling [105].

In 2010, a systematic review did not show evidence of diet efficacy in colicky infants and found limited data of symptoms after the reintroduction of CM protein [106]. In contrast, two years later another systematic review, analysed eleven randomised control trials, considered of high quality, and concluded that both breast-fed and formula-fed colicky infants benefited from a CM elimination diet [107].

In recent years, gastrointestinal symptoms and crying were analysed in several studies testing HFs. In 2014, Vandenplas et al. explored the effect of two casein eHFs in 72 formula-fed infants with symptoms suspected to be related to CMA [97]. The percentage of infants crying for at least 3 h/day decreased from 43.5% at recruitment to 11.6% after one month on the diet (−31.9%, *p* < 0.0001), with a greater difference in the group taking the thickened formula vs. the non-thickened one (−38.3% vs. −25.7%) and in infants who had a positive CM challenge (65%) compared to those with a negative one (−47.1% vs. −17.1%) [97].

In 2018, the Cochrane review on dietary modification for infantile colic included 15 randomized control trials, with a total of 1121 infants recruited, and concluded that 25% of infants with moderate to severe symptoms had a significant reduction in crying time on a CM-free diet compared to the group with intact CM protein intake [108]. However, most studies had a small number of infants and a significant risk of bias.

We prospectively recruited 47 infants on a CM-free diet due to persistent unexplained gastrointestinal symptoms. In 19 (40%) cases, the symptoms improved or resolved when on the diet and, in one third of cases, reoccurred on challenge. The majority of these infants were prescribed an eHF [109].

Symptoms, such as vomiting, regurgitation, and crying, can decrease and disappear because of the natural evolution of these disorders. Likewise, gastroesophageal symptoms may reoccur with standard formulas (with intact proteins and normal lactose content) not only for immune mechanisms, but also for increased fermentation and prolonged gastric emptying time [94].

Myoelectrical abnormalities and gastric dysrhythmia were documented in children with CMA with intact protein intake [110] and appeared to have been mediated by chemokines released by mast cells and eosinophils, and by increased mast cell density next to submucosal nerve endings [106]. In sensitized infants, CM induced bradygastria and delayed gastric emptying, which, in turn, may exacerbate GER and induce vomiting and pain [110].

A CM-free diet reduces mast cell infiltration, and normalizes immune-nerve interactions and motor function [16]. At the same time, HFs reduce symptoms by accelerating gastric emptying [91,94].

Experience showed that pHF may also reduce infant colic when CMA is not implicated [99]. In one study, enrolling 267 colicky babies, a whey-based pHF, containing fructo- and galacto-oligosaccharides and reduced lactose content, showed a significant decrease in crying episodes after two weeks of diet as compared to the group of infants fed a standard formula [111]. Of note, many pHF combined protein hydrolysate with reduced lactose content, prebiotic oligosaccharides, and structured lipids with a high proportion of sn-2-position β-palmitate, which decreased the formation of calcium soaps and stool consistency. Thus, it was difficult to discern which component of the formula was responsible for the clinical effect and reduction of crying. There are no randomized clinical trials performed on infant colic that demonstrate the efficacy of partially hydrolysed proteins as a single change in the formula. For this reason, although the usefulness of pHF in alleviating functional gastrointestinal disorders has been suggested, the available studies are still insufficient to draw evidence-based recommendations [98,112,113].

If colic is associated with other allergic signs and symptoms or a family history of atopy, a trial with an eHF for 2 to 8 weeks may be considered a suitable option to investigate the pathogenetic role of CMA [6,104]. A step- by step approach to infants with persistent unexplained regurgitation, vomiting, and crying was recently proposed to help clinicians in managing these infants and deciding time and type of diet intervention, including HF [94].

## 7. Conclusions

Commercial HF vary in terms of source of protein, degree of hydrolysis, the content of lactose and additional components. Before starting a CM-free diet, clinicians should consider the severity, frequency, and persistence of symptoms, the age of the patient, the growth, the cost of the HF, and the proof of efficacy. If the diet is beneficial, a challenge should be scheduled first to prove the diagnosis of CMA and later to test tolerance acquisition in order to avoid an unnecessary protracted diet. Regurgitation, vomiting, and crying are common symptoms in infants. They occur because of functional gastrointestinal disorders and many other conditions. The symptoms are more persistent and severe in infants with CMA and GERD and are often associated with the involvement of other organs. In these conditions, dietary intervention and HF may be of benefit, both as a result of immune and motor mechanisms. Determining which infants should start taking eHF is still challenging, primarily due to the lack of positive allergy tests or specific biomarkers.

## Figures and Tables

**Table 1:** Nutrient composition of hydrolysed and soy formulas during infancy and the follow-on period.

	Infant Formula		Follow-On Formula	
	Minimum	Maximum	Minimum	Maximum
Energy content	60 kcal/100 mL (250 kJ/100 mL)	70 kcal/100 mL (293 kJ/100 mL)	60 kcal/100 mL (250 kJ/100 mL)	70 kcal/100 mL (293 kJ/100 mL)
PROTEIN				
hydrolysed formulas	1.86 g/100 kcal	2.8 g/100 kcal	1.86 g/100 kcal	2.8 g/100 kcal
soy formulas	2.25 g/100 kcal	2.8 g/100 kcal	2.25 g/100 kcal	2.8 g/100 kcal
Taurin		12 mg/100 kcal		12 mg/100 kcal
L-carnitin	1.2 mg/100 kcal	-	-	-
LIPIDS	4.4 g/100 kcal	6.0 g/100 kcal	4.4 g/100 kcal	6.0 g/100 kcal
Linoleic acid	500 mg/100 kcal	1200 mg/100 kcal	500 mg/100 kcal	1200 mg/100 kcal
Alfa-linolenic acid	50 mg/100 kcal	100 mg/100 kcal	50 mg/100 kcal	100 mg/100 kcal
DHA	20 mg/100 kcal	50 mg/100 kcal	20 mg/100 kcal	50 mg/100 kcal
TRANS fats	-	3% of total lipid content	-	3% of total lipid content
erucic acids	-	1% of total lipid content	-	1% of total lipid content
Choline	25 mg/100 kcal	50 mg/100 kcal	-	-
Inositol	4 mg/100 kcal	40 mg/100 kcal	-	-
Phospholipids		2 g/L		2 g/L
Carbohydrates	9 g/100 kcal	14 g/100 kcal	9 g/100 kcal	14 g/100 kcal
Pre-cooked or gelatinised starch	-	2 g/100 mL and 30% of total CHO content		-
Sucrose (only for hydrolysed formulas)	-	20% of total CHO content	-	20% of total CHO content
Glucose (only for hydrolysed formulas)	-	2 g/100 kcal	-	2 g/100 kcal
Fructo/galacto-oligosaccharides	-	0.8 g/100 mL	-	0.8 g/100 mL
MINERALS (for 100 kcal)				
Sodium (mg)	25	60	25	60
Potassium (mg)	80	160	80	160
chloride (mg)	60	160	60	160
Calcium (mg)	50	140	50	140
Phosphorous (mg)				
hydrolysed formulas	25	90	25	90
soy formulas	30	100	30	100
Magnesium (mg)	5	15	5	15
Iron (mg)				
hydrolysed formulas	0.3	1.3	0.6	2
soy formulas	0.45	2	0.9	2.5
Zinc (mg)				
hydrolysed formulas	0.5	1	0.5	1
soy formulas	0.75	1.25	0.75	1.25
Copper (μg)	60	100	60	100
Iodine (μg)	15	29	15	29
Selenium (μg)	3	8.6	3	8.6
Manganese (μg)	1	100	1	100
Molybdenum (μg)	-	14	-	14
Fluoride (μg)	-	100	-	100
VITAMINS (for 100 kcal)				
Vitamin A (μg -RE)	70	114	70	114
Vitamin D (μg)	2	3	2	3
Thiamine (μg)	40	300	40	300
Riboflavin (μg)	60	400	60	400
Niacin (mg)	0.4	1.5	0.4	1.5
Pantothenic acid (mg)	0.4	2	0.4	2
Vitamin B_6_ (μg)	20	175	20	175
Biotin (μg)	1	7.5	1	7.5
Folate (μg-DFE)	15	47.6	15	47.6
Vitamin B_12_ (μg)	0.1	0.5	0.1	0.5
Vitamin c (mg)	4	30	4	30
Vitamin k (μg)	1	25	1	25
Vitamin e (mg α-tocoferolo)	0.6	5	0.6	5

**Table 2:** Main trials evaluating safety and efficacy of different hydrolysed formulas in the treatment of CMA.

References	Type of Study	Subjects	Type of Formula	Intervention/ Follow-Up Duration	Outcomes	Results
Niggemann 2001 [33]	Multicentric RCT	*N* = 73 infants (median age, 5.7 months) with atopic dermatitis and CMA	EHWF vs. AAF	6 months	Severity of eczema (SCORAD) and growth (length, weight-for-length) measured as median at 3 and 6 months in each group	Both AAF and eHF resulted in a significant clinical improvement; AAF resulted in improved growth compared with eHF
Niggemann 2008 [34]	Multicentric RCT	*N* = 77 infants aged <12 months with suspected CMA	EHWF vs. AAF	6 months	Severity of eczema (SCORAD), allergic manifestation, growth (z-score for length, body weight, and head circumference at 28, 60, 90, and 180 days), adverse effects	No significant differences in growth measurements or allergy symptoms; SCORAD decrease in AAF group
Berni Canani 2017 [35]	Multicentric RCT	*N* = 65 infants aged 5–12 months, with strongly suspected CMA, or healthy controls	EHWF vs. AAF vs. healthy controls	12 months	Growth (z-score for body weight, length/height and head circumference at 3, 6 and 12 months	At 12 months, no significant difference in weight z-scores
Isolauri 1995 [36]	RCT	*N* = 45 infants (mean age: 6 months) with atopic dermatitis and CMA	EHWF vs. AAF	9 months	Growth (body weight and length), severity of eczema (mean SCORAD)	In both groups, atopic eczema improved significantly. Growth was adequate in both groups, though promoted only in AAF infants
Lasekan 2006 [31]	Randomized, blinded, prospective trial	*N* = 65 healthy infants	Partially hydrolysed rice protein-based formula fortified with lysine and threonine vs. standard intact cow’s milk protein-based formula	16 weeks	Growth, tolerance and plasma biochemistries	The two study groups had comparable growth, tolerance, and plasma biochemistry, despite some differences in amino acid profiles
Agostoni 2007 [32]	Randomized, prospective, comparative, unblinded trial	*N* = 160 infants fully breast- fed during the first 4 months of life and diagnosed with CMA within 6 months of age	Soy formula, eHF, hydrolysed rice-based formula vs. breastfed infants	6–12 months of age	growth indices	Infants fed hydrolysed products showed a trend toward higher weight-for-age z-score increments in the 6- to 12-month period
Reche 2010 [43]	Prospective open, randomized clinical study	*N* = 92 infants with IgE-mediated CMA	hydrolysed rice-based formula vs. EHF	24 months	Clinical tolerance	Both formulas were well tolerated. Growth parameters were similar between the two study groups
Vandenplas 2014 [44]	Prospective trial	*N* = 40 infants with CMA	Extensively hydrolysed rice-based formula	6 months	hypoallergenicity and safety	Symptoms significantly decreased in the first month of intervention; catch-up to normal weight gain as of the first month as well as a normalization of the weight-for-age, weight-for length, and BMI z-scores within the 6-month study period
Vandenplas 2014 [45]	Prospective trial	*N* = 39 infants with a confirmed CMA	Extensively hydrolysed rice-based formula	One month	Tolerance and growth	Extensively hydrolysed rice-based formula was tolerated by more than 90% of children with proven CMPA; and weight and length gains were normal
Rzehak 2011 [46]	Prospective, randomized, double-blind trial	*N* = 1840 full-term neonates with atopic heredity	pHF-W, eHF-W, eHF-C, CMF, breastfed	16 weeks and 10 years	differences in body mass index (BMI) over the first 10 years of life	No significant differences in BMI trajectories were shown between the study groups at 10 years of age

**Table 3:** Recommendations from current guidelines.

	DRACMA—2010	ESPGHAN—2012	EAACI—2014	BSACI—2014
Partially hydrolysed formula		Not recommended for infants with CMA	Not regarded as safe for patients with CMA	They are not hypoallergenic and therefore should not be used for the treatment of suspected or proven CMA or diagnostic exclusion diets
Amino acid formula	-Anaphylaxis -FPIES -Allergic eosinophilic oesophagitis -Heiner’s syndrome -eHF non-responder patients	-Breast-fed with severe symptoms (not evidence based) -Formula-fed with severe or life-threatening symptoms both IgE- and non-IgE-mediated -In atopic children >2 years with multiple food allergies or in cases of eosinophilic disorders of the digestive tract -If there is no improvement within 2 weeks with eHF	-Severe growth faltering -Severe symptoms -Non-IgE-mediated syndromes, such as food protein-induced enterocolitis and eosinophilic gastro-enteropathies	-Multiple food allergies-Severe cow’s milk allergy -Allergic symptoms or severe atopic eczema when exclusively breastfed -Severe forms of non IgE-mediated cow’s milk allergy (EoE, enteropathies, and FPIES) -Faltering growth -Reacting to or refusing to take eHF
Cow’s milk protein based eHF	-Immediate GI allergy -Asthma and rhinitis -Acute urticaria or angioedema -Atopic dermatitis -GERD -Cow’s milk protein-induced enteropathy -functional gastrointestinal disorders (constipation and colic) -CM protein induced gastroenteritis and proctocolitis	First choice in formula-fed infants with proven CMA	First choice in formula-fed infants with proven CMA	Forms of milk protein allergies not included in the indications for AAF
Soy formula Soy hydrolysed formula	CM eHF, rather than soy formula (SF), are well tolerated in infants with IgE-mediated CMA, but to a lesser extent in those with non-IgE-mediated CMA; SF should not be considered in infants <6 months of age	Once tolerance to soy protein is established in: -Infants >6 months who do not accept the bitter taste of a CM eHF, if high cost is a limiting factor, or if there are strong parental preferences (e.g., a vegan diet).	Soy formulas may be useful provided that nutritional evaluation regarding the phytate and phytoestrogens content is considered, and they cannot be recommended before 6 months of age	Soy protein formula could be considered in children >6 months of age once tolerance to soy protein is established
Rice hydrolysed formula	Equivalent to CM eHF in countries where they are available	-Infants refusing or not tolerating a CM eHF or in vegan families	Further research is needed to compare these formulas with CM eHF	Rice milk should not be used under 4.5 years of age due to its natural inorganic arsenic content
Prebiotics and probiotics	More RCTs need to be conducted to elucidate whether probiotics are useful	No evidence that they have a role in treatment of CMA	Currently, probiotic supplements cannot be recommended for the management of food allergies	Evidence of preventative or therapeutic activity for food allergy is lacking

**Table 4:** Hydrolysed formulas: pros and cons.

Type of Formula	Results	Pros	Cons
CM-based pHF	May improve symptoms of FGIDs Conflicting efficacy in prevention of allergy and eczema Improved gastric emptying vs. standard formulas	Often combined with pro/prebiotic components, reduced lactose and modified fat Better palatability compared to eHF and AAF	Not suitable to treat CMA Absence of long-term follow-up data Limited controlled trials Higher cost vs. standard formulas
CM based eHF	Efficacy in 90% of patients with CMA Accelerates gastric emptying vs. pHF and vs. standard formulas	first choice for CMA treatment, except for anaphylaxis, EoE, and severe CMA May improve symptoms of FGIDs Better absorption of peptides vs. amino-acids	Much higher cost vs. standard formulas Absence of lactose in most eHF Poor palatability and possible effect on taste development
Amino-acid formula	Efficacy in 100% of infants with CMA Efficacy in 75–90% of patients with EoE	First choice treatment for severe CMA, anaphylaxis, and EoE in infants	Much higher cost vs. eHF and vs. standard formulas Absence of lactose Poor palatability and possible effect on taste development
Rice-based eHF	Reported efficacy in the treatment of CMA in selected subjects	Second choice treatment for CMA, except for anaphylaxis, EoE, and severe CMA	Arsenic content should be limited and labelled Different amino-acid profile compared to CM based formulas Limited number of studies (mostly on IgE-mediated CMA)
Soy-based infant formulas	Possible efficacy in selected subjects with CMA	Low cost	Not recommended in the first 6 months of life and in infants with gastrointestinal symptoms Possible allergy to soy Concerns related to phytoestrogens and transgenic modified soybean

## Data Availability

Not applicable.

## Abbreviations

CM	Cow’s milk
FA	Food allergies
CMA	Cow’s milk allergy
FPIAP	Food protein-induced allergic proctocolitis
FPIES	Food protein-induced enterocolitis syndrome
EoE	Eosinophilic esophagitis
HF	Hydrolysed formulas
pHF	Partially hydrolysed formulas
eHF	Extensively hydrolysed formulas
AAF	Amino acid formulas
RCTs	Randomised controlled trials
GER	Gastroesophageal reflux
GERD	Gastroesophageal reflux disease
